# Supplementary material for: Global sampling decline erodes science potential of natural history collections
Source: Nat Commun. 2025 Oct 20;16:9255. doi: 10.1038/s41467-025-64303-3 (PMC12537823; doi:10.1038/s41467-025-64303-3)
Supplement: Supplementary file 1 — Supplementary Information [file 41467_2025_64303_MOESM1_ESM.pdf]

Supplementary Information

Global Sampling Decline Erodes Science Potential of Natural History Collections

Authors:

Owen Forbes

Andrew G. Young

Peter H. Thrall

Table of Contents

Supplementary Note 1. Specimens, Unique Species & Spatial Extent: 1800-2024............2

Supplementary Note 3. Data Cleaning – GBIF Contemporary Records 5

Supplementary Note 4. GBIF Database Snapshots – Records by Collection Year – Data Cleaning...9

Supplementary Note 5. Methodological Caveats 13

Supplementary Note 6. GBIF Database Snapshots – Growth Over Time....................... 15

Supplementary Note 7. ARIMA Forecasts – In-sample Validation 19

Supplementary Note 8. Chordata – Detailed Taxonomic Splits (Mammals, Birds, Fish) .. 22

Supplementary Note 9. Chordata – Specimens Per Year by Continent 23

Supplementary Note 10. Plantae – Specimens Per Year by Continent 24

Supplementary Note 11. Arthropoda – Specimens Per Year by Continent 25

Supplementary Note 12. Global Map of Natural History Collection Institutions Contributing to GBIF 26

Supplementary References 27

Supplementary Note 1. Specimens, Unique Species & Spatial Extent: 1800-2024

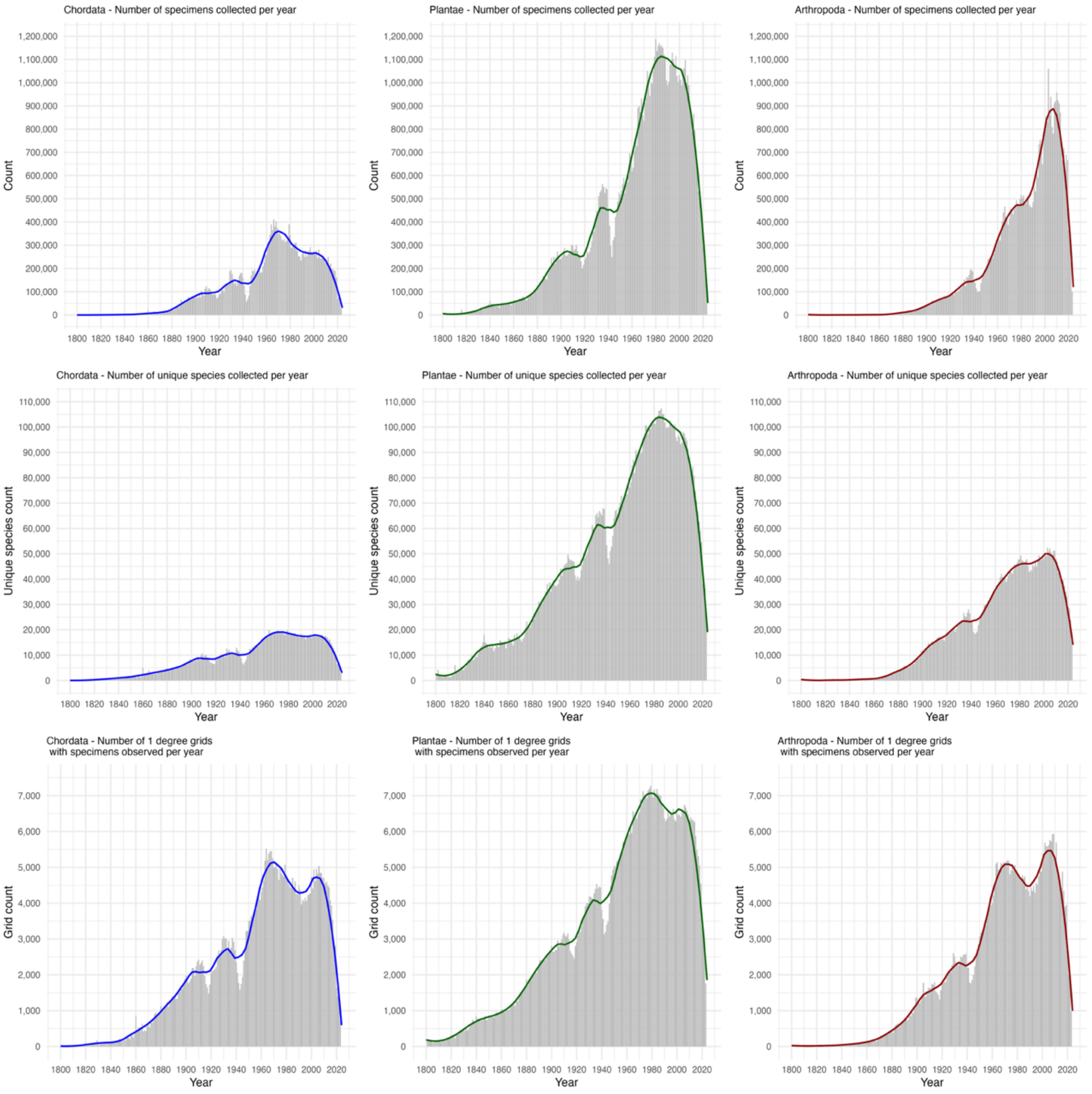

Supplementary Figure 1: GBIF Records – Number of Specimens, Unique Species and Spatial Extent for Chordata, Arthropoda and Plantae (1800-2024).

Top row = Chordata; Middle row = Arthropoda; Bottom row = Plantae. Left column = Number of specimens per year; Middle Column = Number of unique species per year; Right Column = Spatial Extent (Number of 1-degree grid cells with specimens, per year). Coloured lines indicate LOESS curves (locally estimated scatterplot smoothing) with span = 0.2.

Supplementary Note 2. Results by Continent – All Three Taxa Combined

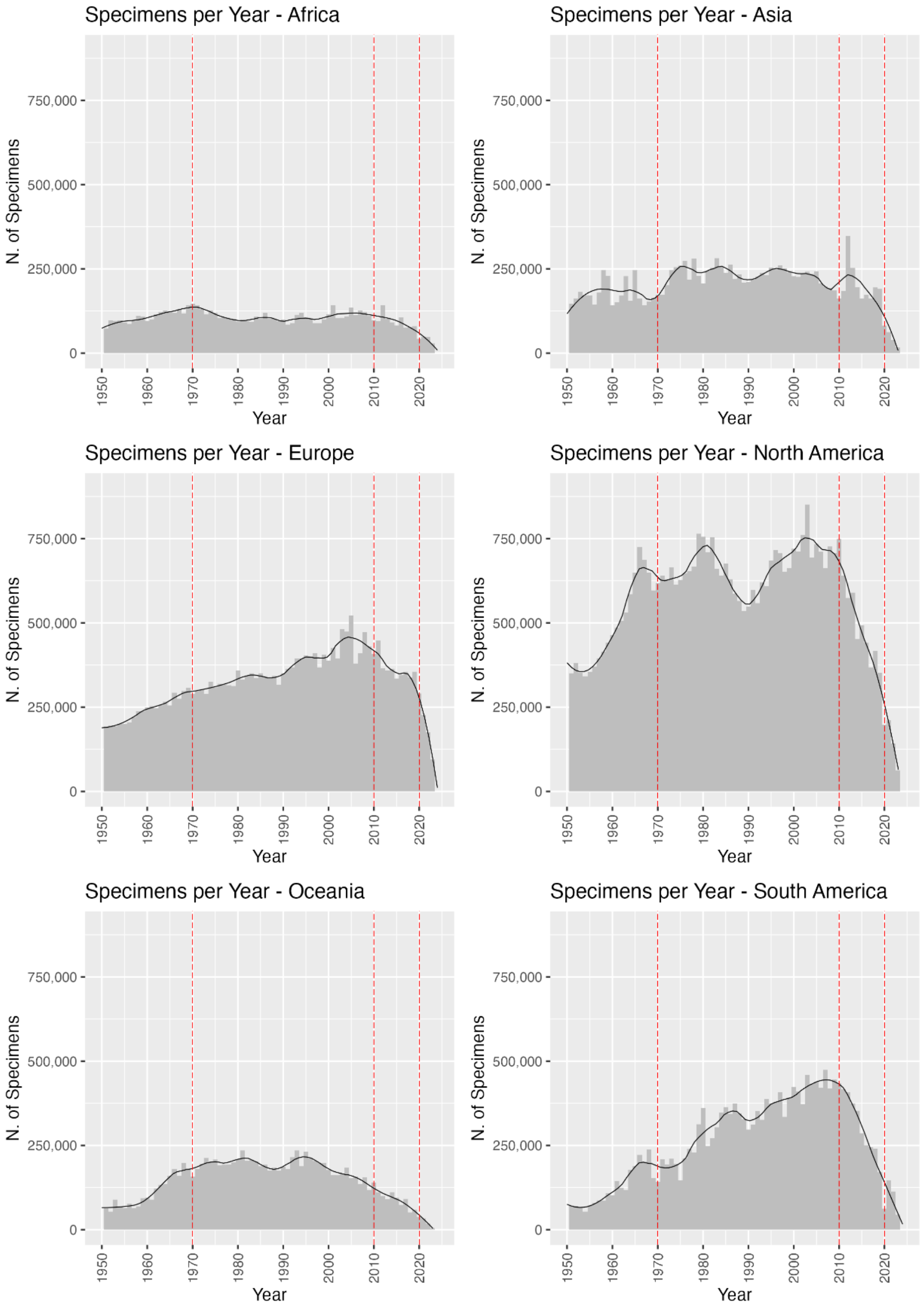

Supplementary Figure 2. GBIF Records – Number of Specimens by Continent for Chordata, Arthropoda and Plantae (combined), 1950-2024.

Vertical dotted lines demarcate the reference periods used for visualising trends in Figure 4, contrasting recent collecting (2010-2019) relative to the broad peak period (1970-2009). Black lines indicate LOESS curves (locally estimated scatterplot smoothing) with span = 0.2.

Supplementary Note 3. Data Cleaning – GBIF Contemporary Records

We filtered the institution-level datasets of contemporary records within each taxon, removing instances where a dataset had greater than 3.0 standard deviations above the mean on the log scale for records per dataset per collection year, across all years, and relative to datasets in that specific collection year. This process resulted in the removal of 235,187 records (0.87%) from 1 of 1,974 datasets for Chordata; 4,832,798 records (9.56%) from 4 of 3,244 total datasets for Arthropoda; and 1,408,072 records (1.58%) from 2 of 2,402 total datasets for Plantae. Further details on data cleaning for contemporary records are available in Supplementary Figure 3 and Supplementary Table 1. The relatively large number of records removed for Arthropoda were concentrated between 2012-2014 and were largely driven by confirmed duplication of one large dataset, as a result of duplicated uploads from the original institution (CBG Guelph) and another aggregator platform (INSDC EMBL-EBI; Supplementary Table 1).

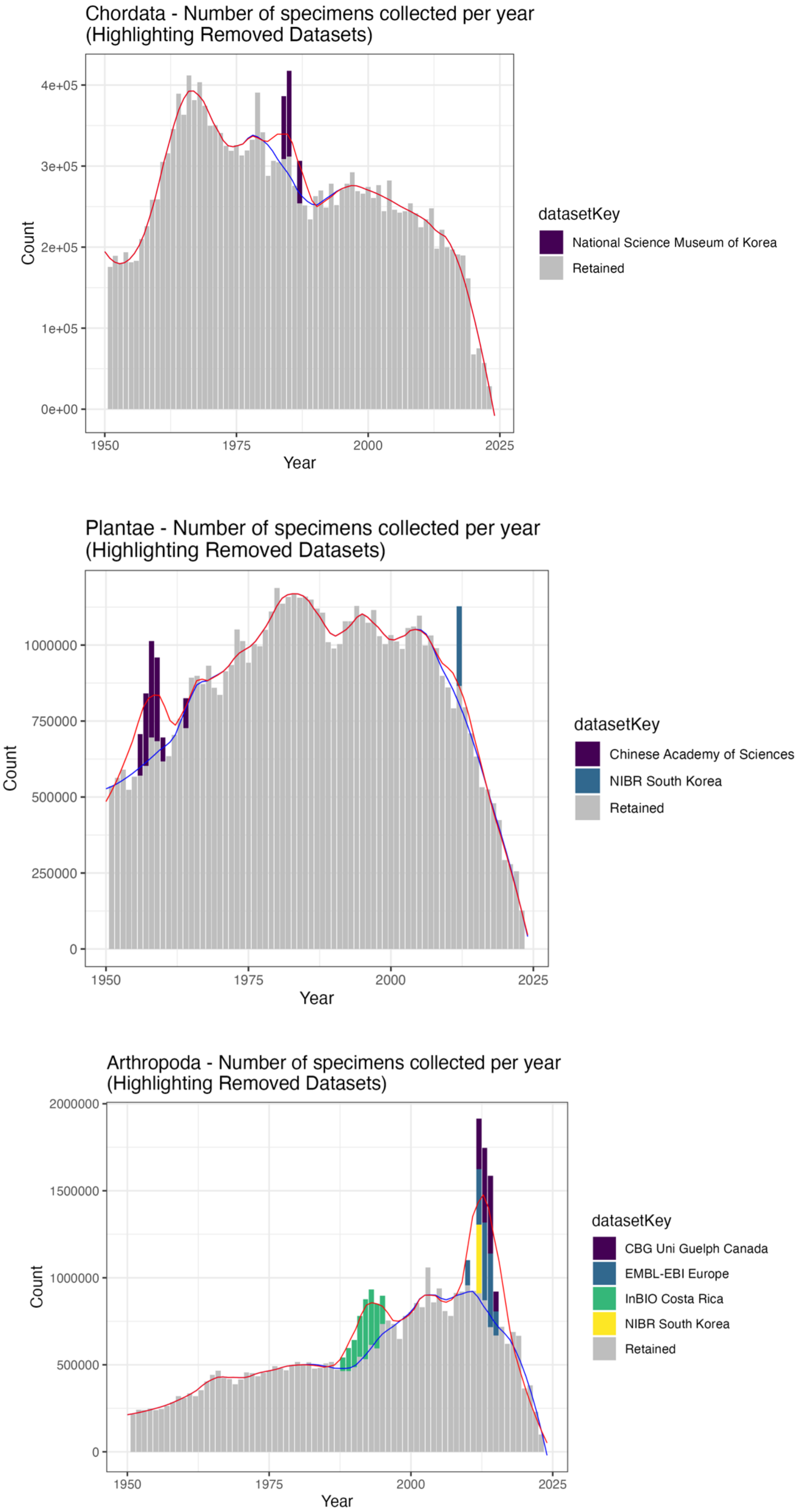

Supplementary Figure 3. GBIF contemporary records - specimens per collection year, highlighting removed anomalous records by taxon, dataset and collection year.

Supplementary Table 1. GBIF contemporary data - removed anomalous records, by dataset and year of collection.

Taxon	Year	Dataset	N. Records removed	Log Z-score (Total)	Log Z-score (Annual)
**Chordata**					
	1984	NSMK Fish	77256	3.37	3.15
	1985	NSMK Fish	105613	3.5	3.23
	1987	NSMK Fish	52318	3.2	3.04
**Plantae**					
	1956	CAS	136878	3.27	3.3
	1957	CAS	238943	3.5	3.46
	1958	CAS	317807	3.61	3.53
	1959	CAS	275653	3.55	3.56
	1960	CAS	78773	3.05	3.04
	1964	CAS	97514	3.14	3.04
	2012	NIBR Flora & Fauna	262504	3.53	3.19
**Arthropoda**					
	1988	InBIO Insecta	77450	3.22	3.04
	1989	InBIO Insecta	133082	3.44	3.29
	1990	InBIO Insecta	156757	3.5	3.36
	1991	InBIO Insecta	233297	3.66	3.42
	1992	InBIO Insecta	344941	3.82	3.6
	1993	InBIO Insecta	319127	3.79	3.51
	1994	InBIO Insecta	256977	3.7	3.42
	1995	InBIO Insecta	163754	3.52	3.2
	2010	INSDC EMBL-EBI	143983	3.47	3.1
	2012	NIBR Flora & Fauna	394705	3.88	3.45
	2012	INSDC EMBL-EBI	318939	3.79	3.37
	2012	CBG UoG	289808	3.75	3.33
	2013	INSDC EMBL-EBI	446051	3.93	3.46

Taxon	Year	Dataset	N. Records removed	Log Z-score (Total)	Log Z-score (Annual)
**Arthropoda**	2013	CBG UoG	429527	3.91	3.44
	2014	INSDC EMBL-EBI	422499	3.91	3.49
	2014	CBG UoG	448377	3.93	3.52
	2015	INSDC EMBL-EBI	138840	3.45	3.11
	2015	CBG UoG	114684	3.38	3.03

Log Z-score (Total) = Number of log scale standard deviations above the mean count of records per year, relative to all datasets and years; Log Z-score (Annual) = Number of log scale standard deviations above the mean count of records per year, relative to other datasets in that year.

NSMK Fish = National Science Museum of Korea Fish Resources; CAS = Contributions of Plant Specimen Data inside China, Chinese Academy of Sciences; InBIO Insecta = Insecta of Costa Rica (InBIO); INSDC EMBL-EBI = INSDC Sequences, European Nucleotide Archive (EMBL-EBI); NIBR Flora & Fauna = South Korean National Institute of Biological Resources Flora & Fauna; CBG UoG = Centre for Biodiversity Genomics - Canadian Specimens, University of Guelph.

Supplementary Note 4. GBIF Database Snapshots – Records by Collection Year – Data Cleaning

For historical snapshot data used for forecast models, prior to data cleaning there were multiple instances of unusually large spikes for records in particular collection years, which were later removed from GBIF. We conducted data cleaning to address these transient spikes, in addition to removal of other anomalies from contemporary records as described above. After iterative testing we used thresholds of 3.1 log scale standard deviations for filtering Plantae and Chordata, and 3.6 for Arthropoda. A higher threshold was used for Arthropoda to account for the larger variability in annual record counts, particularly for collection years 2012 - 2014. This resulted in a removal of 0.7% of records for Chordata, 13.9% for Arthropoda (of which 7.8% were in datasets already flagged during cleaning of contemporary records, and 5.9% in collection years 2012 - 2014), and 1.5% of records for Plantae. These thresholds were selected to be conservative, removing a small number of highly anomalous datasets while maximising the data retained. Further details are available in Supplementary Figures 4 – 9.

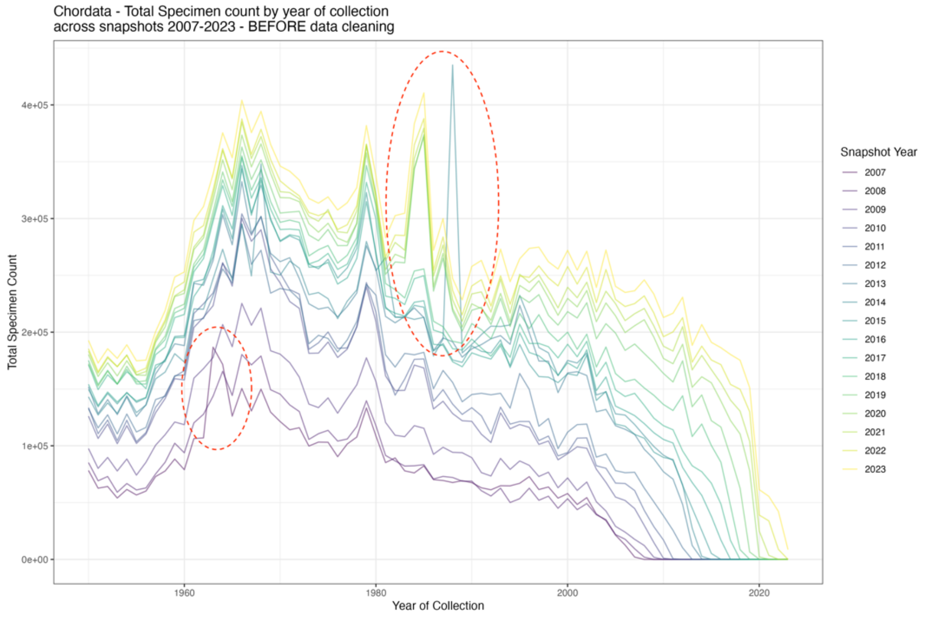

Supplementary Figure 4. Chordata specimen count (1950-2020) across snapshots (2007-2023) before data cleaning, with anomalies highlighted in dashed red lines.

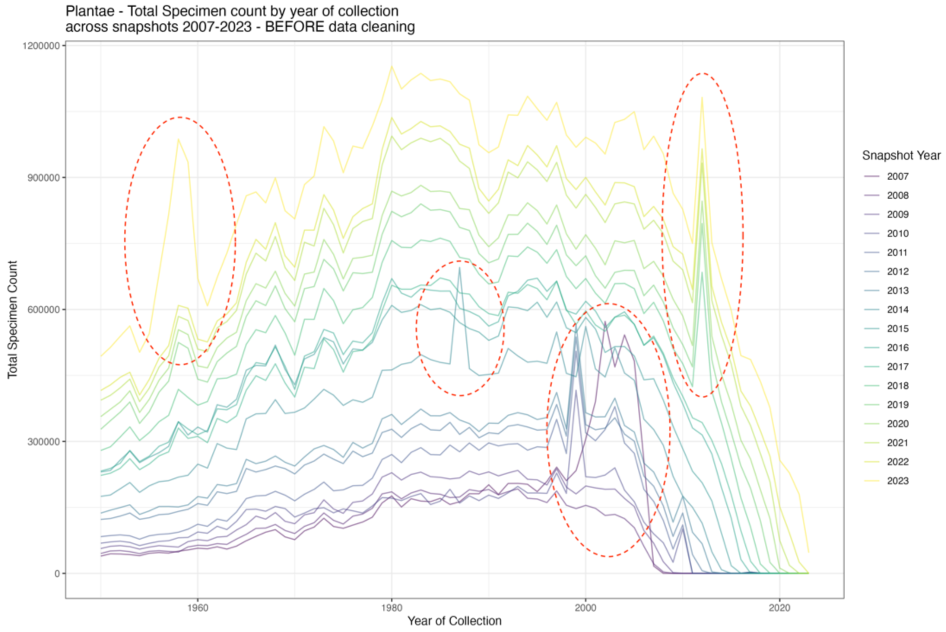

Supplementary Figure 5. Plantae specimen count (1950-2020) across snapshots (2007-2023) before data cleaning, with anomalies highlighted in dashed red lines.

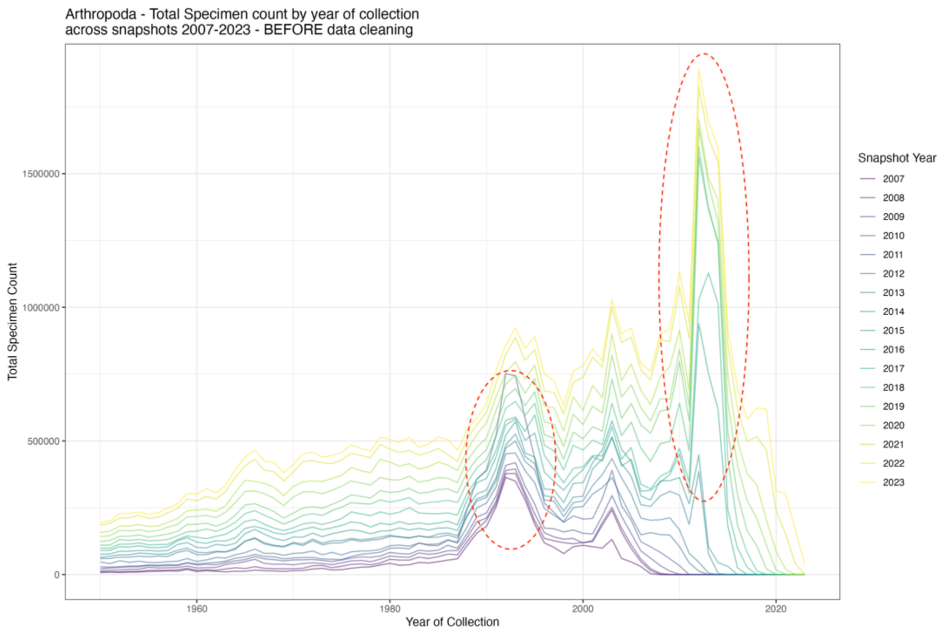

Supplementary Figure 6. Arthropoda specimen count (1950-2020) across snapshots (2007-2023) before data cleaning, with anomalies highlighted in dashed red lines.

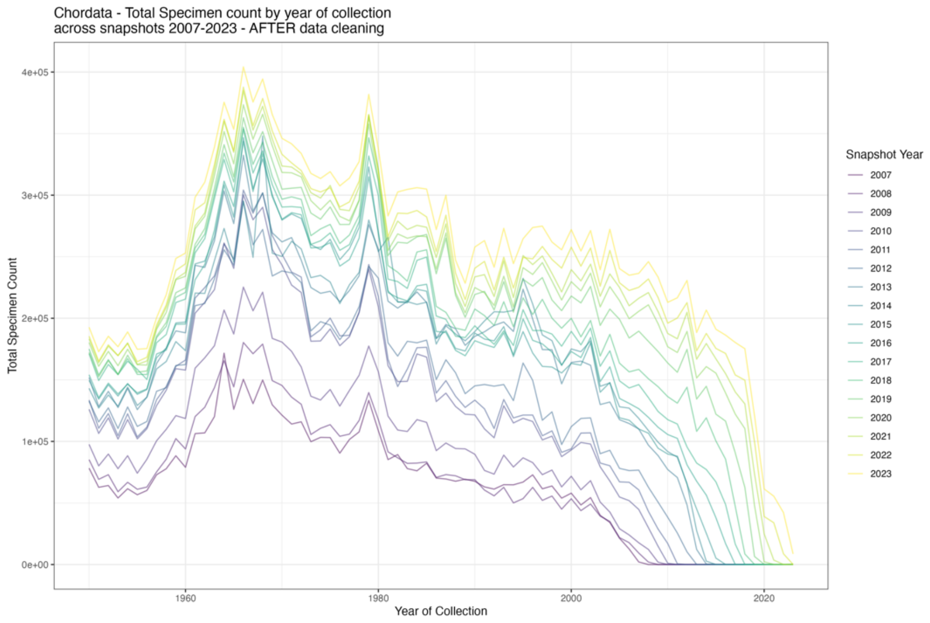

Supplementary Figure 7. Chordata specimen count (1950-2020) across snapshots (2007-2023) after data cleaning with a threshold of 3.1 standard deviations on the log scale.

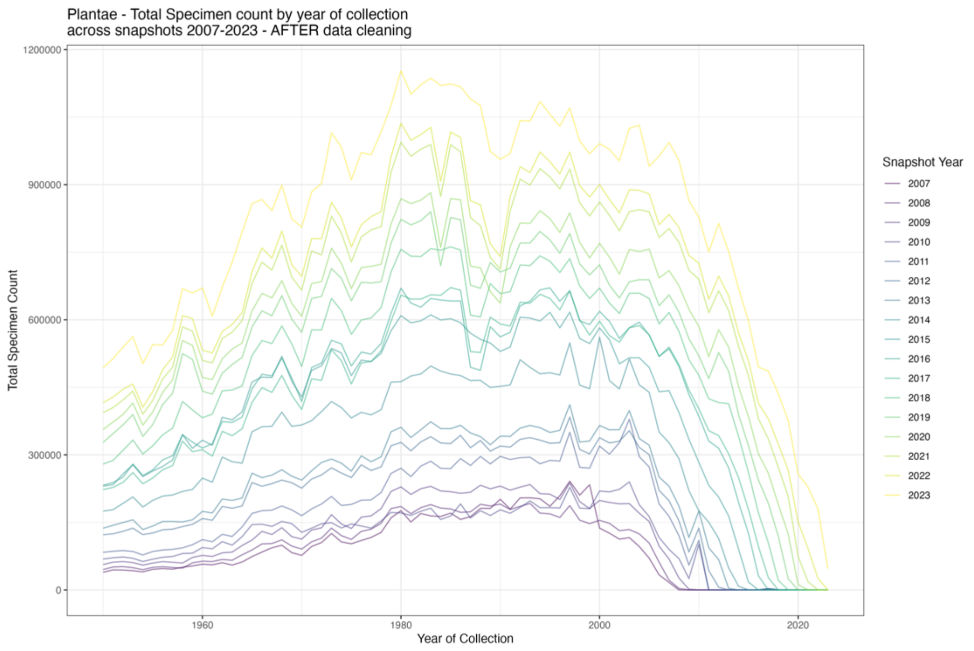

Supplementary Figure 8. Plantae specimen count (1950-2020) across snapshots (2007-2023) after data cleaning with a threshold of 3.0 standard deviations on the log scale.

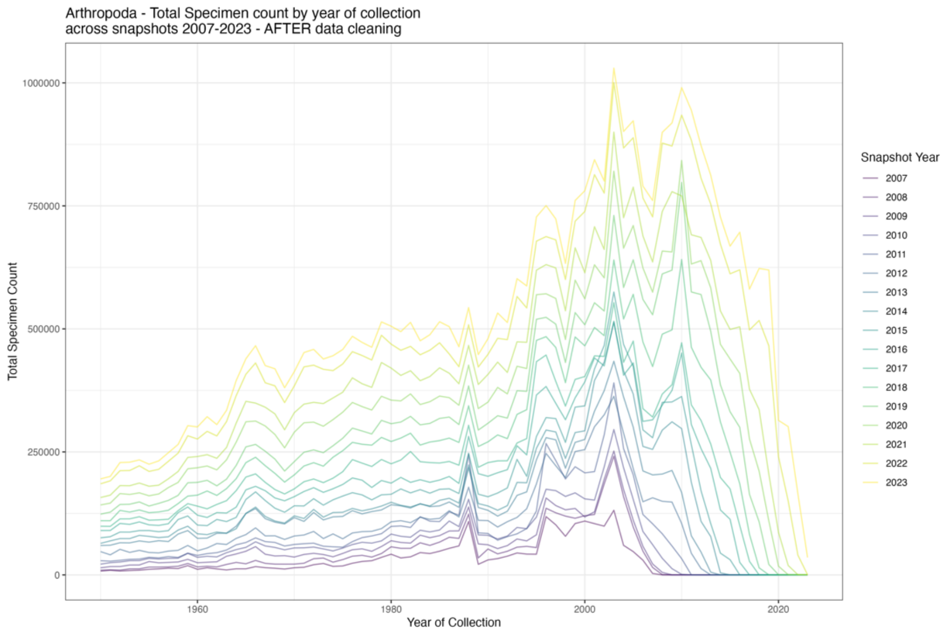

Supplementary Figure 9. Arthropoda specimen count (1950-2020) across snapshots (2007-2023) after data cleaning with a threshold of 3.6 standard deviations on the log scale.

Supplementary Note 5. Methodological Caveats

There are several methodological caveats to acknowledge, regarding challenges we encountered during the processes of assessing how cataloguing and databasing delays may affect estimated annual specimen counts, and our inability to undertake exhaustive data cleaning given the scale and complexity of these datasets.

One significant challenge was estimating the effect of backlogs in specimen preparation, identification, and database accessioning. The impact is likely most substantial for Arthropoda, given ongoing high-volume collection activities and large numbers of uncatalogued specimens. The absence of databasing and accession dates for GBIF records limited our ability to disentangle three distinct processes: (1) actual declines in specimen collecting activity, (2) time lags between collection and local databasing, and (3) delays between local databasing and GBIF contribution. These processes vary across institutions and taxonomic groups, with arthropods typically experiencing longer delays due to their diversity and identification complexity. To address this limitation, we implemented forecasting analyses using historical GBIF database snapshots (2007-2019), which consistently support our conclusion that observed declines represent genuine collection trends rather than databasing artifacts. Variability across manual and automated data upload pipelines to GBIF also introduces complexity in interpreting recent trends.

To address data anomalies, we implemented a cautious approach to data cleaning. We identified and addressed high-level data quality concerns, removing major anomalous instances from 7 out of 7,620 datasets across the three taxa (Supplementary Materials Section 3; Supplementary Table 1). This iterative process removed a small fraction of clearly anomalous records while retaining as much data as possible. It is important to note that our cleaning process was not exhaustive and further data quality issues likely remain to be

resolved at institutional or aggregator levels, including GBIF's ongoing quality assurance processes.

Supplementary Note 6. GBIF Database Snapshots – Growth Over Time

Looking at average at year-on-year growth in records by year of collection, our analysis shows that recent records since GBIF snapshots started (2007 onwards) are growing at a similar rate to older records for Plantae and Chordata. Arthropoda particularly demonstrate a trend towards higher growth in records for recent collection years, particularly for collection years 2010 – 2020. For all three taxa, there is greater uncertainty in the average growth rates for recent years of collection (Supplementary Figures 10 - 12).

Even after data cleaning (Supplementary Materials Sections 4 – 5), there are remaining instances where a decline in specimen record counts was observed in some snapshot years for certain collection year ranges. This is reflective of GBIF's own data quality and checking processes, as well as institutions modifying and updating their databases over time. As addressed in the Discussion, given the scale of the data and the limited available information on specific dataset attributes, our data cleaning processes were not intended to be exhaustive, and only focus on extreme anomalies which would distort high level inferences regarding trends over time.

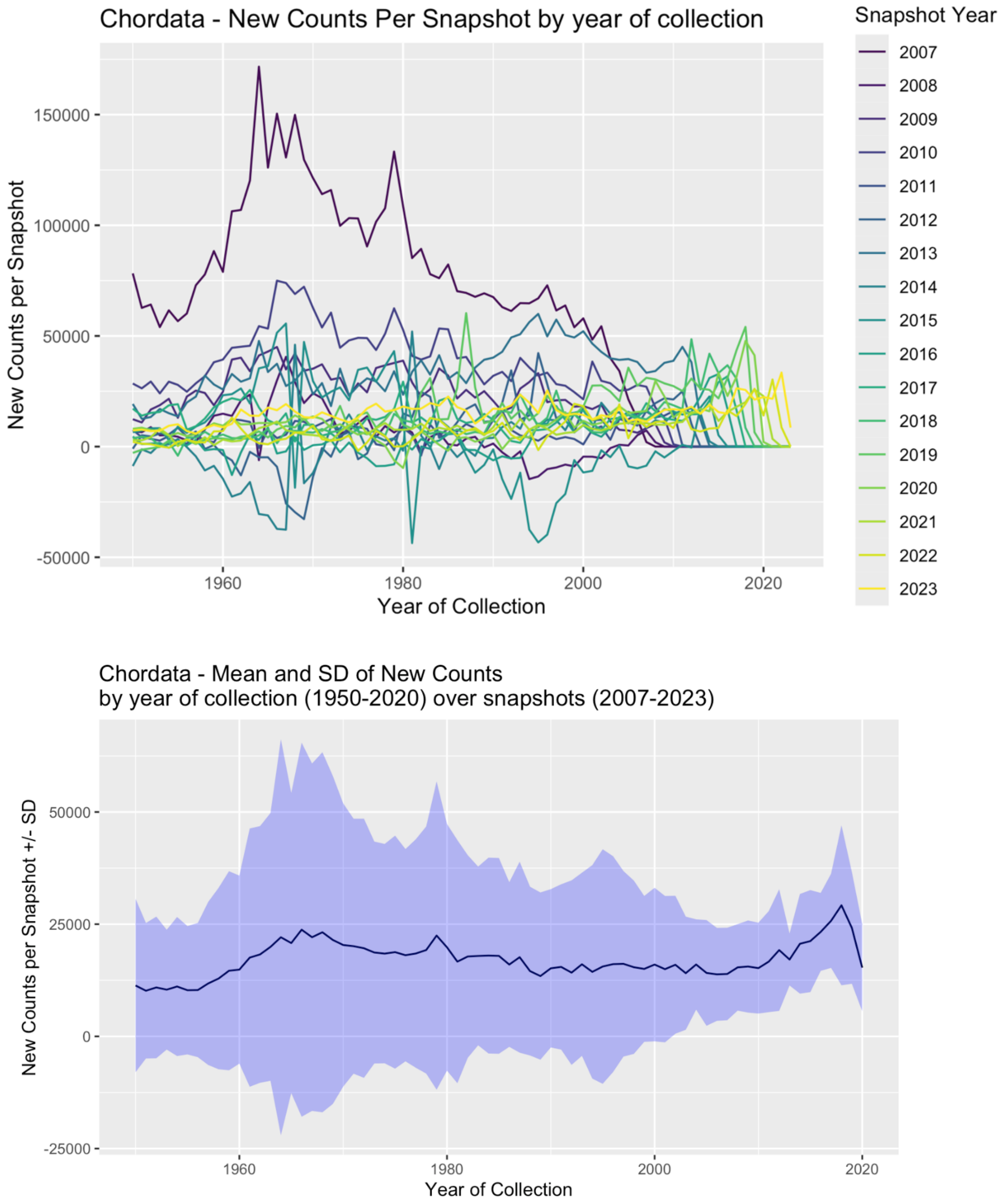

Supplementary Figure 10. Chordata – Change in number of specimens per collection year (1950-2020) over snapshot years (2007-2023) after data cleaning.

Top panel: Change in each snapshot year. Bottom panel: Mean and standard deviation of change over all snapshot years.

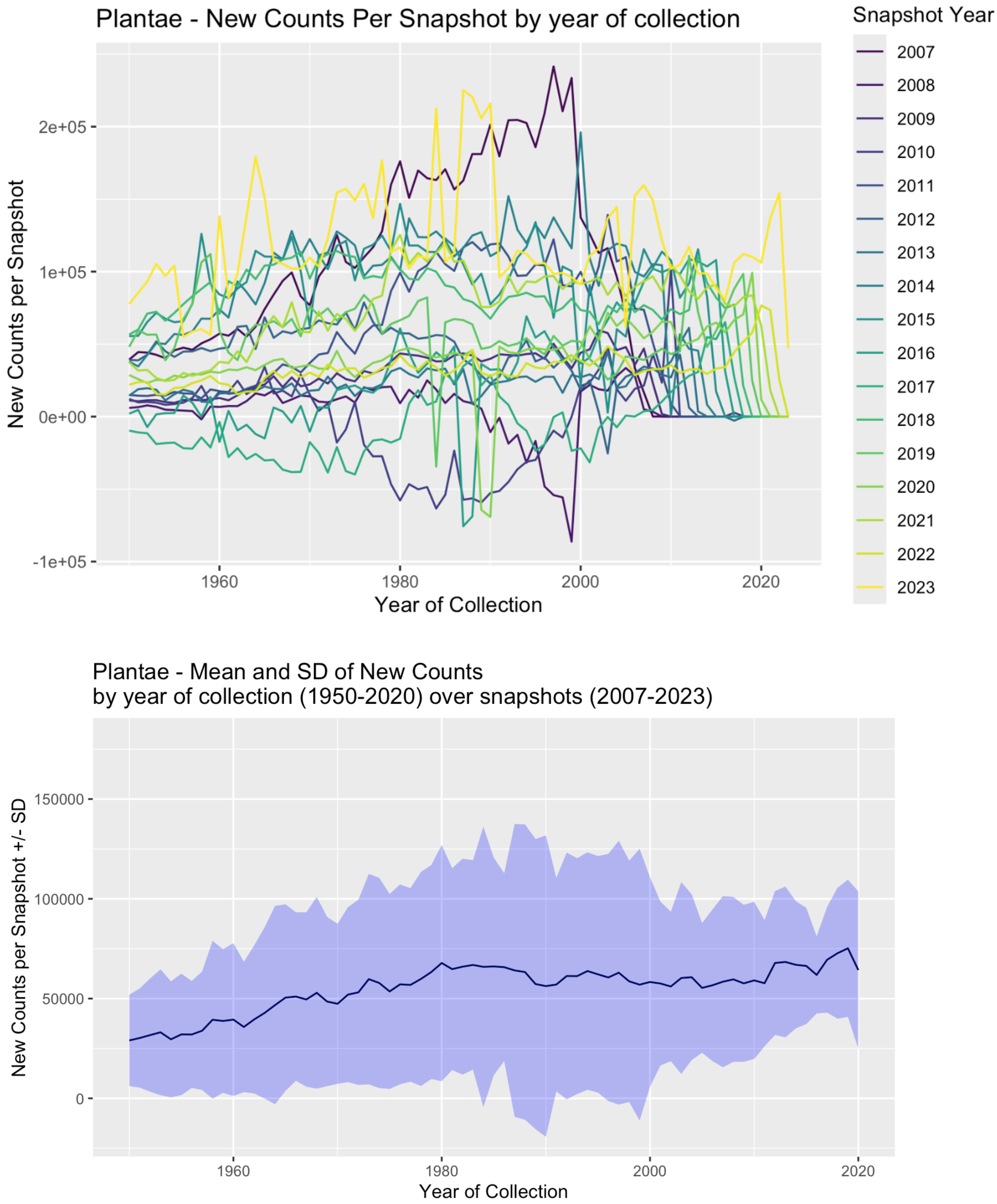

Supplementary Figure 11. Plantae – Change in number of specimens per collection year (1950-2020) over snapshot years (2007-2023) after data cleaning.

Top panel: Change in each snapshot year. Bottom panel: Mean and standard deviation of change over all snapshot years.

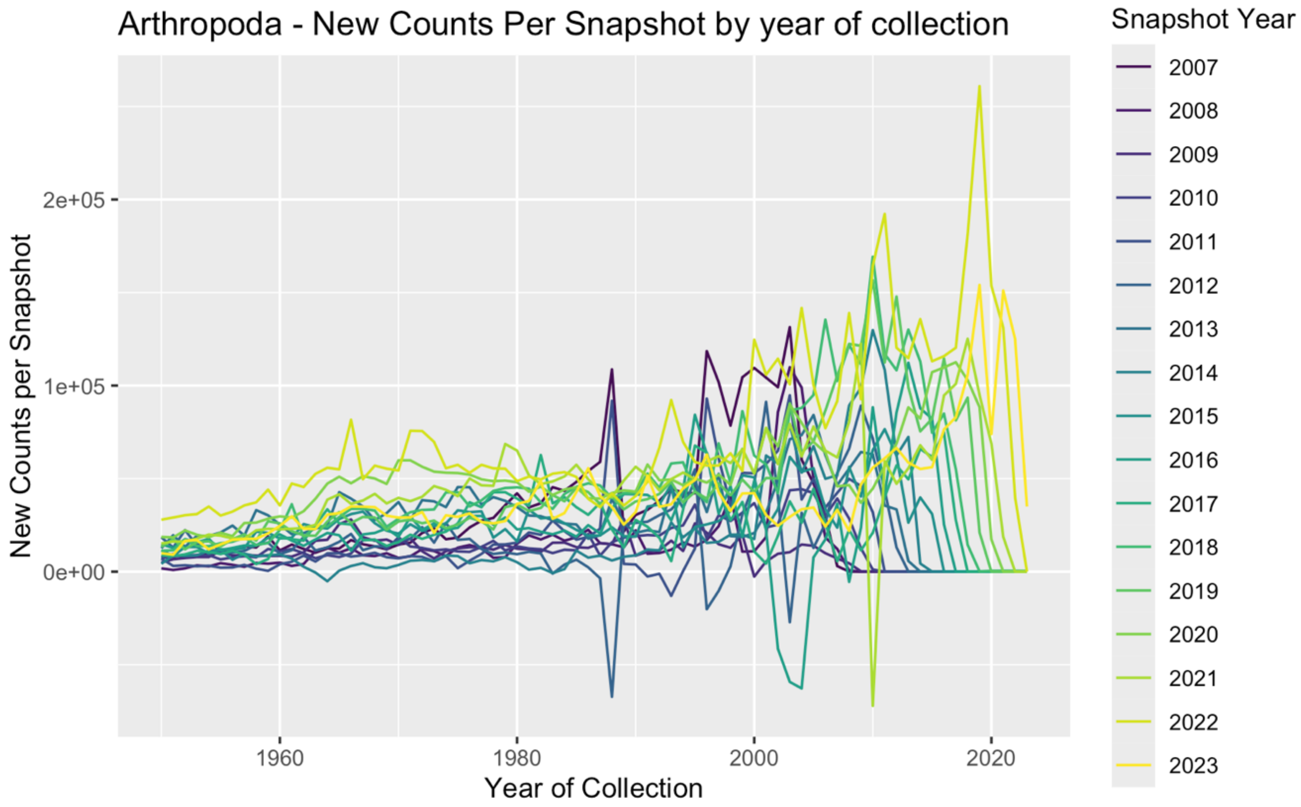

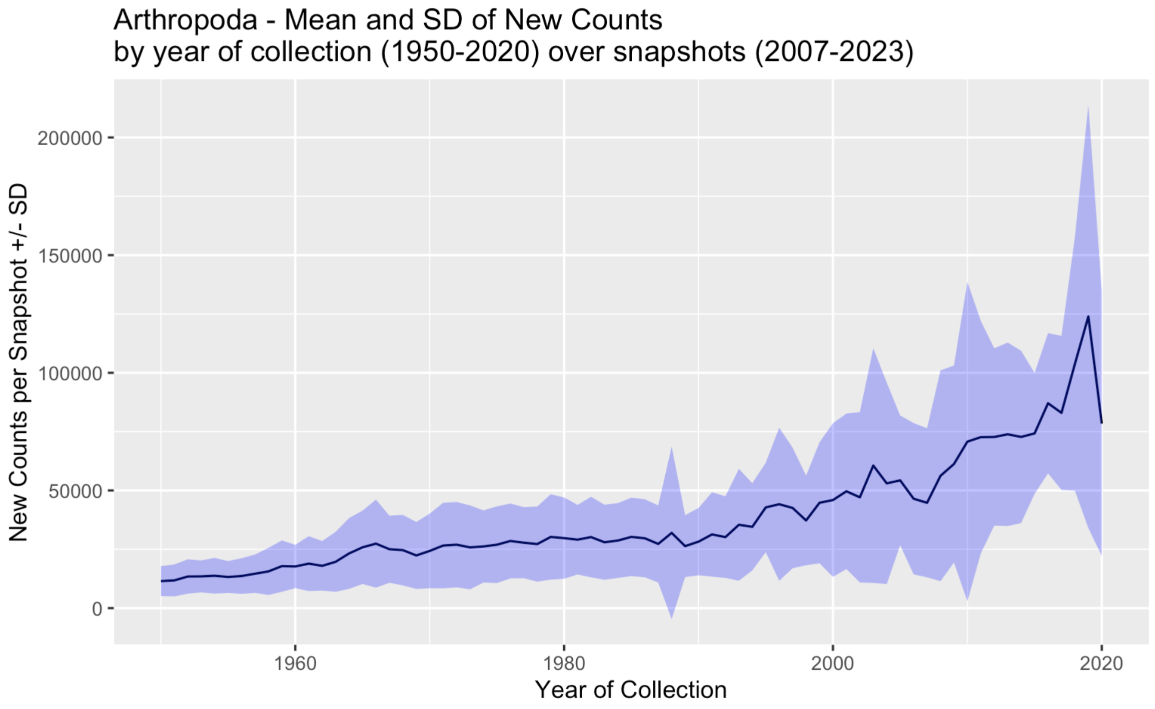

Supplementary Figure 12. Arthropoda – Change in number of specimens per collection year (1950-2020) over snapshot years (2007-2023) after data cleaning.

Top panel: Change in each snapshot year. Bottom panel: Mean and standard deviation of change over all snapshot years.

Supplementary Note 7. ARIMA Forecasts – In-sample Validation

We conducted in-sample validation to assess performance of ARIMA models, and to choose the appropriate order of differencing to use for models in each taxon. Initially we tested automatic selection of differencing order per model based on the correct Akaike Information Criterion (AICc), and found that this process resulted in large discrepancies and inconsistency across forecasts for adjacent years of collection. As a result, we chose to select one order of differencing for each taxonomic group based on the root mean square prediction error (RMSPE) for in-sample validation predictions of counts in 2012, 2017 and 2022. Comparison of root mean squared prediction error indicated that on average, first-order differencing offered the best performance for forecasting models in all three taxonomic groups (Supplementary Table 2), and this was implemented for the sake of consistency and comparability across all forecasting models.

In-sample validation indicated acceptable performance of ARIMA forecasts with first-order differencing, with 95% confidence intervals containing the real observed values at 5-year, 10-year and 15-year in-sample horizons for snapshot years 2012, 2017 and 2022 (Supplementary Figure 13). In-sample predictive performance was stronger for collection years prior to 2012, with higher uncertainty in forecasts for more recent years based on fewer available timepoints to generate forecasts forwards from snapshot year 2007. This greater uncertainty for collection years after 2012 is also evident for future forecasts to 2028 and 2033 (Figure 2).

Supplementary Table 2. Root Mean Square Prediction Error (RMSPE) for ARIMA models at 5-year (2012), 10-year (2017) and 15-year (2022) in-sample validation forecasts, using differencing orders of d = 0, d = 1, and d = 2.

	d = 0	d = 1	d = 2
Chordata			
RMSPE_2012	38002.9	28749.6	77993.0
RMSPE_2017	40175.3	30369.9	131128.8
RMSPE_2022	85773.5	53058.5	179306.1
RMSPE_Mean	54650.6	37392.7	129476.0
Plantae			
RMSPE_2012	289164.1	114342.1	131328.0
RMSPE_2017	117880.7	132565.5	358996.2
RMSPE_2022	273002.6	137475.6	635725.4
RMSPE_Mean	226682.4	128127.7	375349.9
Arthropoda			
RMSPE_2012	212248.3	86517.22	88719.75
RMSPE_2017	110917.3	126944.4	219662.2
RMSPE_2022	261690.1	218845.6	496524.8
RMSPE_Mean	194951.9	144102.4	268302.3

RMSPE_Mean indicates the mean across 2012, 2017 & 2022. d = 0: zero order differencing; d = 1: first-order differencing; d = 2: second-order differencing.

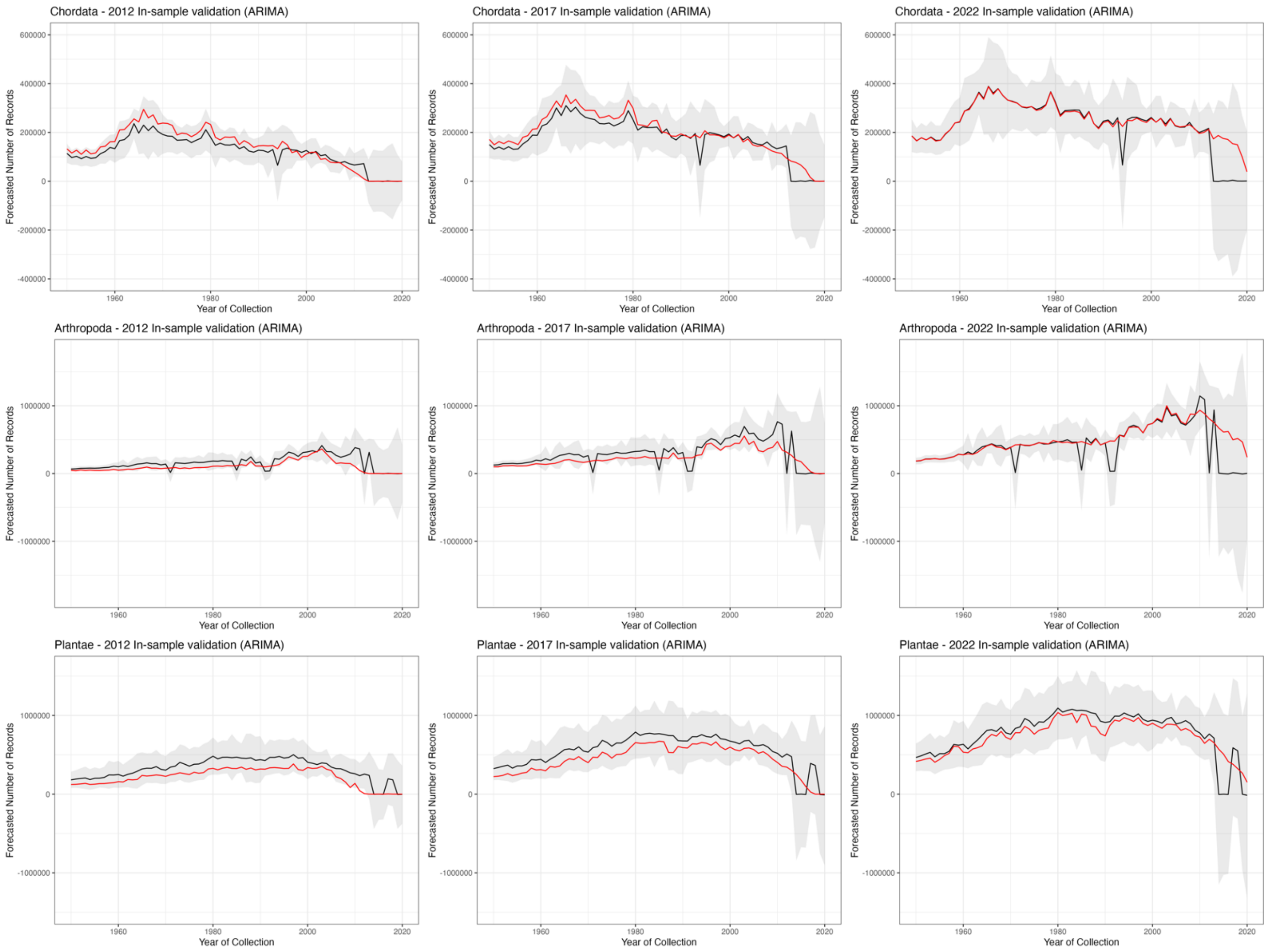

Supplementary Figure 13. In-sample validation of ARIMA forecasts for snapshot years 2012, 2017 and 2022.

Black lines and grey ribbons represent ARIMA forecasts and 95% confidence intervals. Red lines represent actual observed counts by year of collection in each snapshot year. Top row = Chordata; Middle row = Arthropoda; Bottom row = Plantae.

Left column = 2012; Middle column = 2017; Right column = 2022.

Supplementary Note 8. Chordata – Detailed Taxonomic Splits (Mammals, Birds, Fish)

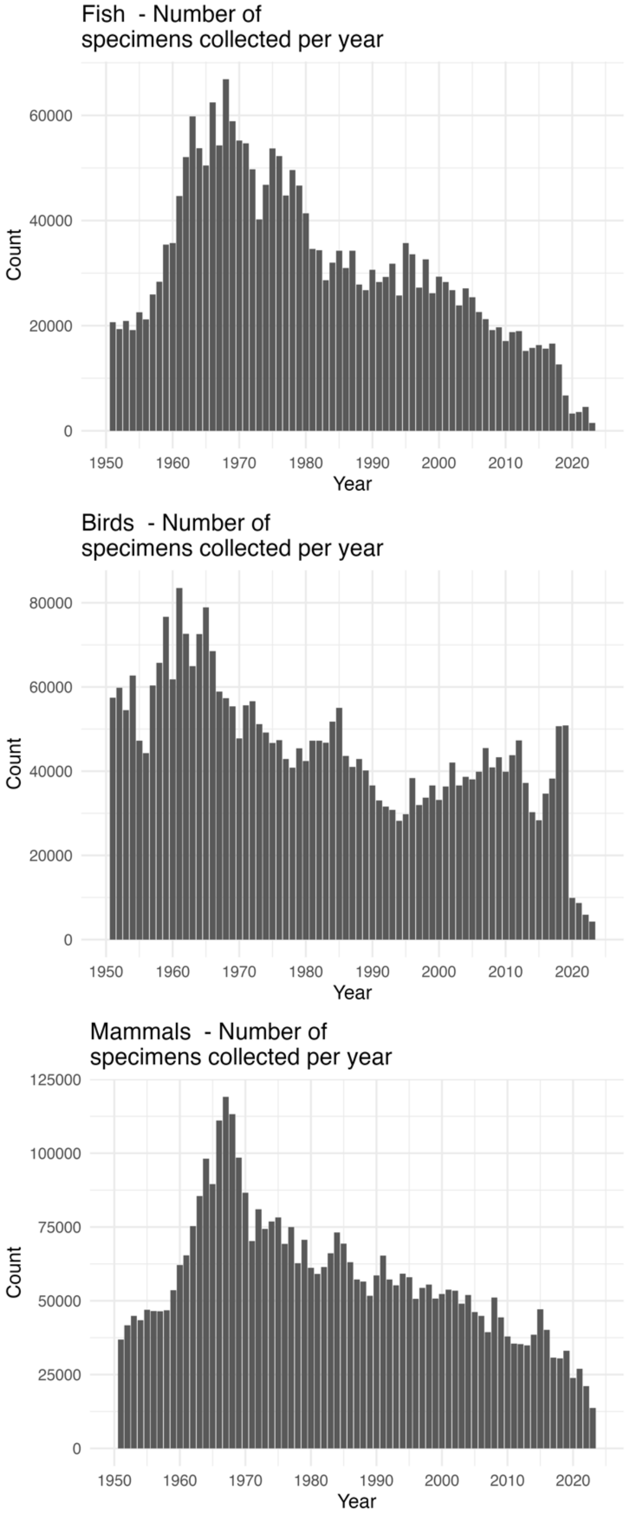

Supplementary Figure 14. Chordata – Specimens per collection year by more detailed taxonomic splits for Fish, Birds and Mammals (1950-2023).

Supplementary Note 9. Chordata – Specimens Per Year by Continent

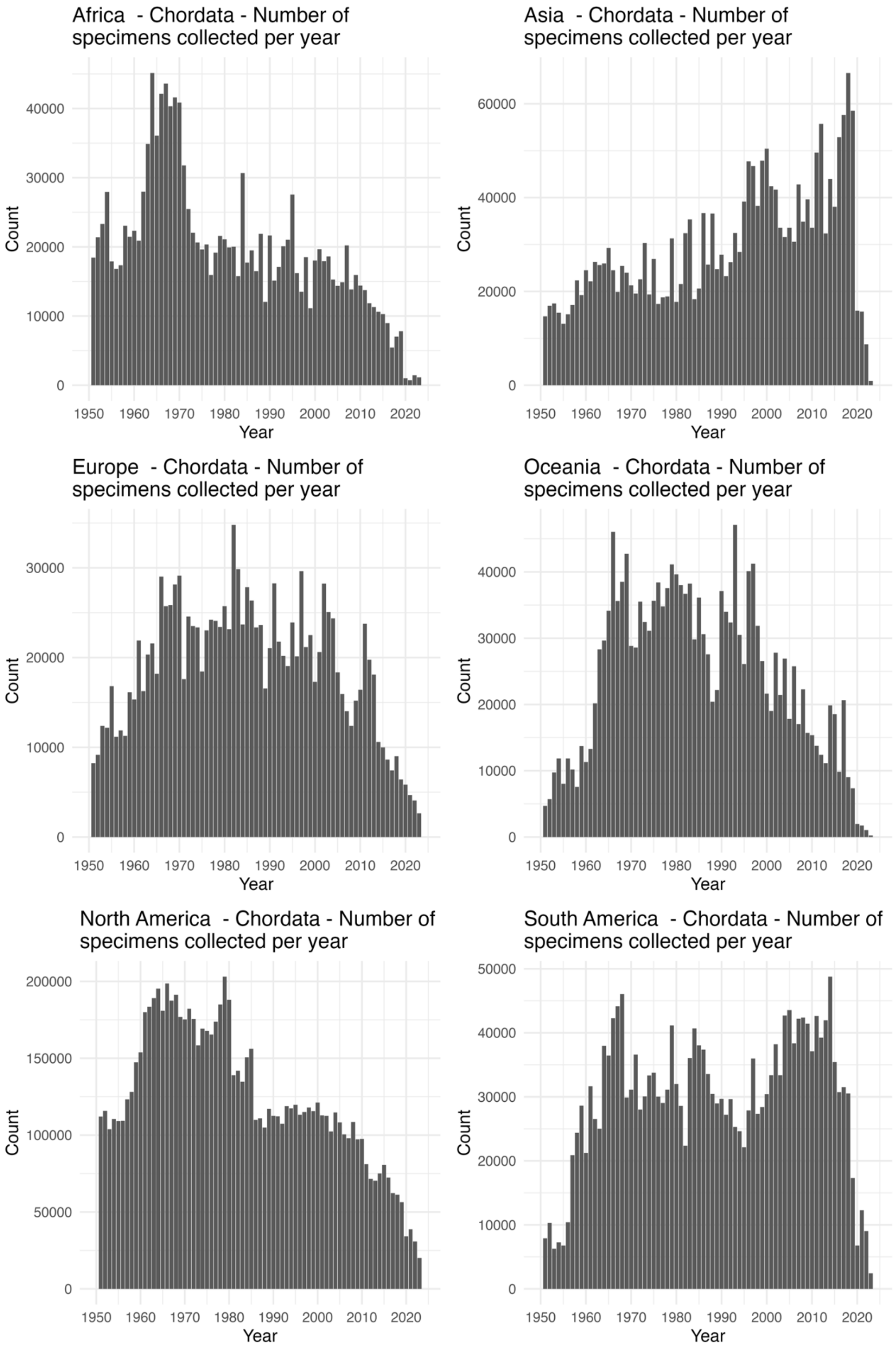

Supplementary Figure 15. Chordata – Specimens per collection year by continent (1950-2023).

Supplementary Note 10. Plantae – Specimens Per Year by Continent

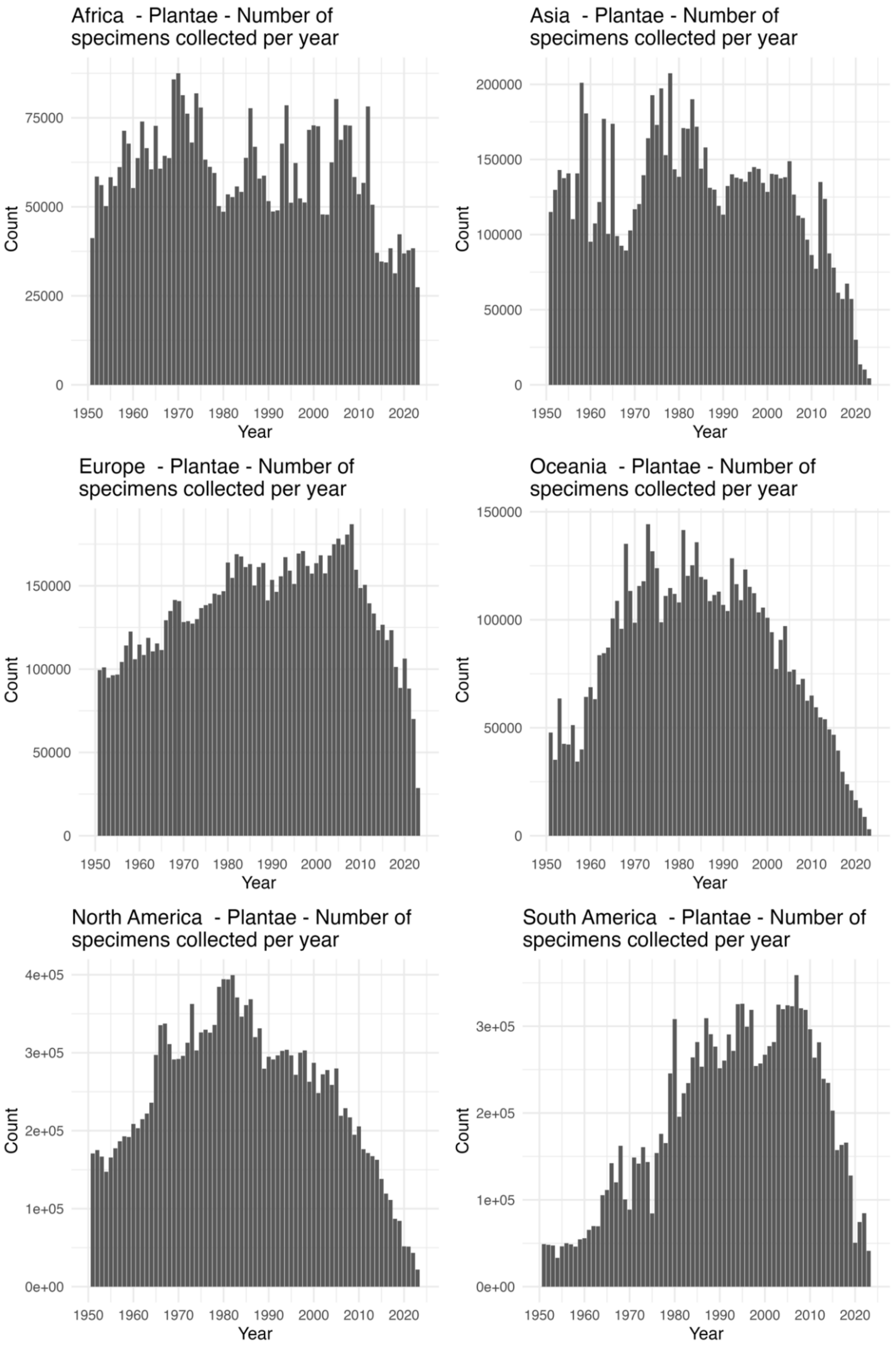

Supplementary Figure 16. Plantae – Specimens per collection year by continent (1950-2023).

Supplementary Note 11. Arthropoda – Specimens Per Year by Continent

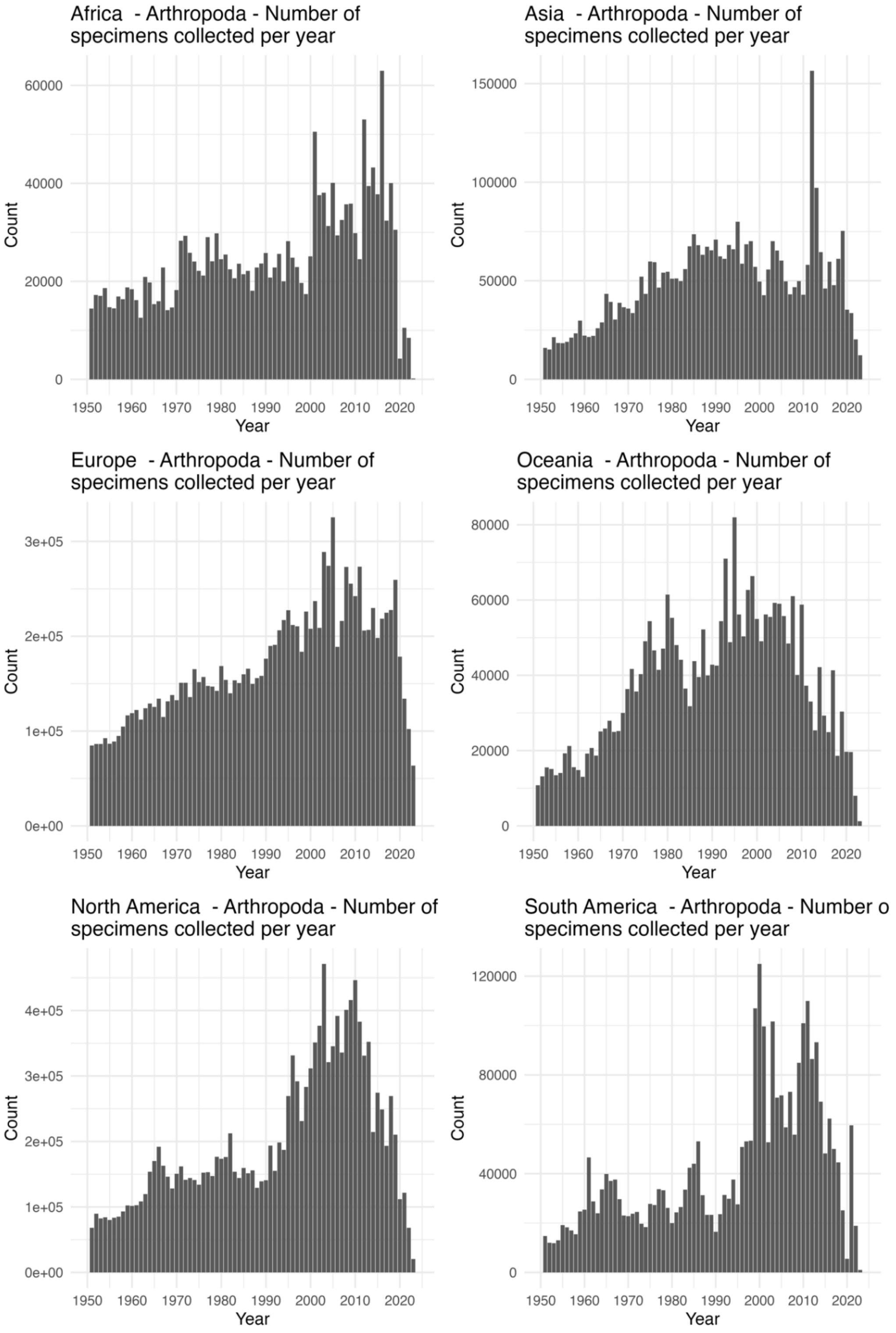

Supplementary Figure 17. Arthropoda – Specimens per collection year by continent (1950-2023).

Supplementary Note 12. Global Map of Natural History Collection Institutions Contributing to GBIF

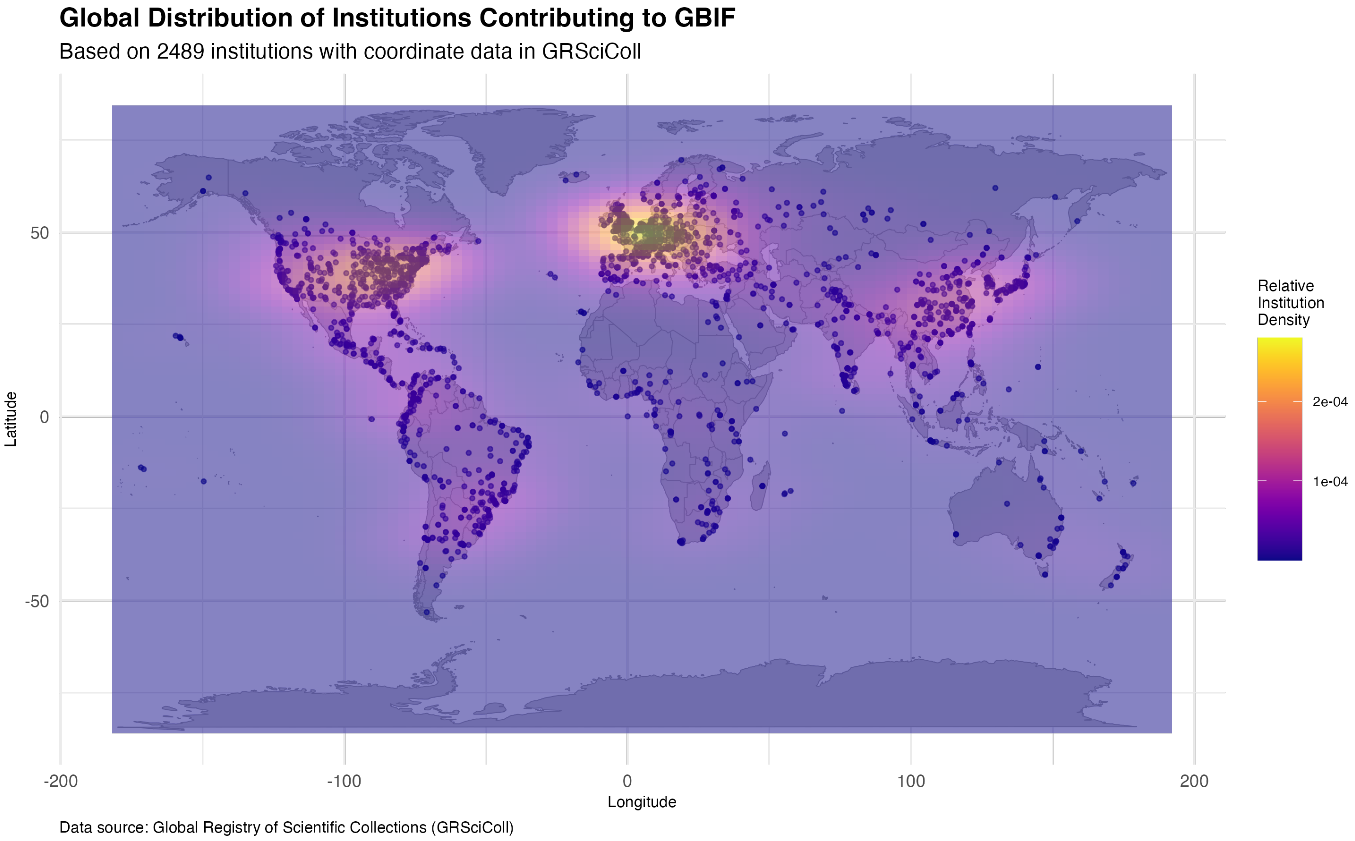

Supplementary Figure 18. Global distribution of institutions contributing specimen data to GBIF.

Blue points represent individual institutions with coordinate data in the Global Registry of Scientific Collections (GRSciColl; n=2,489), while the color gradient indicates relative institution density. The map reveals substantial geographic bias in the distribution of contributing institutions, with particularly high concentrations in North America and Western Europe. This uneven distribution of contributing institutions may influence the observed spatial patterns in specimen collection trends, particularly in regions with fewer contributing institutions. Data source: Global Registry of Scientific Collections[1].

Supplementary References

1. Grosjean, M., Høfft, M., Gonzalez, M. L., Robertson, T., & Hahn, A. (2021). GRSciColl: Registry of Scientific Collections maintained by the community for the community. *Biodiversity Information Science and Standards*, *5*, e74354.
