# Supplementary material for: Global sampling decline erodes science potential of natural history collections
Source: Nat Commun. 2025 Oct 20;16:9255. doi: 10.1038/s41467-025-64303-3 (PMC12537823; doi:10.1038/s41467-025-64303-3)
Supplement: Supplementary file 3 — Reporting Summary [file 41467_2025_64303_MOESM3_ESM.pdf]

nature portfolio

Corresponding author(s): Owen Forbes

Last updated by author(s): Aug 5, 2025

Reporting Summary

Nature Portfolio wishes to improve the reproducibility of the work that we publish. This form provides structure for consistency and transparency in reporting. For further information on Nature Portfolio policies, see our Editorial Policies and the Editorial Policy Checklist.

Statistics

For all statistical analyses, confirm that the following items are present in the figure legend, table legend, main text, or Methods section.

n/a	Confirmed	
☐	☒	The exact sample size (*n*) for each experimental group/condition, given as a discrete number and unit of measurement
☒	☐	A statement on whether measurements were taken from distinct samples or whether the same sample was measured repeatedly
☐	☒	The statistical test(s) used AND whether they are one- or two-sided<br>*Only common tests should be described solely by name; describe more complex techniques in the Methods section.*
☒	☐	A description of all covariates tested
☐	☒	A description of any assumptions or corrections, such as tests of normality and adjustment for multiple comparisons
☐	☒	A full description of the statistical parameters including central tendency (e.g. means) or other basic estimates (e.g. regression coefficient) AND variation (e.g. standard deviation) or associated estimates of uncertainty (e.g. confidence intervals)
☒	☐	For null hypothesis testing, the test statistic (e.g. *F*, *t*, *r*) with confidence intervals, effect sizes, degrees of freedom and *P* value noted<br>*Give P values as exact values whenever suitable.*
☒	☐	For Bayesian analysis, information on the choice of priors and Markov chain Monte Carlo settings
☒	☐	For hierarchical and complex designs, identification of the appropriate level for tests and full reporting of outcomes
☒	☐	Estimates of effect sizes (e.g. Cohen's *d*, Pearson's *r*), indicating how they were calculated

Our web collection on statistics for biologists contains articles on many of the points above.

Software and code

Policy information about availability of computer code

Data collection	*Provide a description of all commercial, open source and custom code used to collect the data in this study, specifying the version used OR state that no software was used.*
Data analysis	Analyses were conducted using the open source statistical software R version 4.3.2, and packages tidyverse v. 2.0.0, sp v. 2.1-3, sf v. 1.0-15 , spdep v. 1.3-3 , rnaturalearth v. 1.0.1 , countrycode v. 1.6.0 , readr v. 2.1.5 , tseries v. 0.10-55 , lubridate v. 1.9.3 , data.table v. 1.15.4 , zoo v. 1.8-12 , scales v. 1.3.0 , purrr v. 1.0.2 , arrow v. 15.0.2.9000 , and forecast v. 8.22.0. Visualisations were generated using ggplot2 v. 3.4 , viridis v. 0.6.5 , ggpubr v. 0.6.0 , and gridExtra v. 2.3. All code used for analyses in this manuscript is publicly available on the associated Zenodo repository.<br><br>All code files for analyses in this manuscript are available in the Zenodo repository with the identifier 10.5281/zenodo.14010665. This repository contains analysis scripts, data processing pipelines, and documentation. To run the analyses, we recommend opening the Quarto '.qmd' files using RStudio, which is freely available and open source IDE software. A README file in the repository provides instructions for reproducing the analyses.

For manuscripts utilizing custom algorithms or software that are central to the research but not yet described in published literature, software must be made available to editors and reviewers. We strongly encourage code deposition in a community repository (e.g. GitHub). See the Nature Portfolio guidelines for submitting code & software for further information.

Data

Policy information about availability of data

All manuscripts must include a data availability statement. This statement should provide the following information, where applicable:

- Accession codes, unique identifiers, or web links for publicly available datasets
- A description of any restrictions on data availability
- For clinical datasets or third party data, please ensure that the statement adheres to our policy

All data used for analyses in this work are publicly available. There are no restrictions on data availability.
Given the large file sizes of the specimen record datasets, instead of uploading to the Zenodo repository we direct users to GBIF directly to access the specimen record datasets. GBIF data exports are available at the following links:
GBIF.Org User. Occurrence Download - Chordata. https://www.gbif.org/occurrence/download/0016915-240425142415019 (2024) doi:10.15468/DL.CNRXZT.
GBIF.Org User. Occurrence Download - Plantae. https://www.gbif.org/occurrence/download/0016914-240425142415019 (2024) doi:10.15468/DL.8MX5ZK.
GBIF.Org User. Occurrence Download - Arthropoda. https://www.gbif.org/occurrence/download/0016913-240425142415019 (2024) doi:10.15468/DL.23TSXM.

The datasets of GBIF historical database snapshots, used for forecasting analyses, as well as other output datasets used for generating figures, are available in the Zenodo repository with the identifier 10.5281/zenodo.14010665

Research involving human participants, their data, or biological material

Policy information about studies with human participants or human data. See also policy information about sex, gender (identity/presentation), and sexual orientation and race, ethnicity and racism.

Reporting on sex and gender	NA
Reporting on race, ethnicity, or other socially relevant groupings	NA
Population characteristics	NA
Recruitment	NA
Ethics oversight	NA

Note that full information on the approval of the study protocol must also be provided in the manuscript.

Field-specific reporting

Please select the one below that is the best fit for your research. If you are not sure, read the appropriate sections before making your selection.

☐ Life sciences ☐ Behavioural & social sciences ☒ Ecological, evolutionary & environmental sciences

For a reference copy of the document with all sections, see nature.com/documents/nr-reporting-summary-flat.pdf

Ecological, evolutionary & environmental sciences study design

All studies must disclose on these points even when the disclosure is negative.

Study description	We analysed large scale data from the Global Biodiversity Information Facility (GBIF) for specimen-based records of Arthropoda, Chordata, and Plantae. We analysed number of specimens, unique species, and 1-degree grids (spatial extent) per year, for each of these taxonomic groups after data cleaning. We conducted forecasting using autoregressive integrated moving average (ARIMA) models to understand how these trends are likely to evolve into the future, as new records continuing being aggregated into GBIF.
Research sample	We analysed large scale data from the Global Biodiversity Information Facility (GBIF) for specimen-based records of Arthropoda, Chordata, and Plantae. For each taxon we exported all specimen-based records from GBIF on May 6, 2024, and initially filtered the data to keep only those records with valid, non-missing entries for species and year of collection between 1800 - 2024. For spatial analyses, we further filtered each dataset to only include records with valid, non-missing entries for decimal latitude and longitude. For each taxonomic group, we calculated the number of records per collection year, the number of unique species represented per year, and the number of 1-degree grids with records present per year, as a measure of spatial extent.
Sampling strategy	We used all available specimen-based data for these taxonomic groups from GBIF.
Data collection	Data were collected by multiple researchers and institutions around the world between 1800-2024.
Timing and spatial scale	Data were collected between 1800 and 2024, and are widely distributed across the planet.

Data exclusions	Through visual inspection of plots for contemporary records and historical snapshots of the GBIF database, we identified a small number of anomalous instances where the numbers of specimens recorded for an individual institution in certain years of collection were so disproportionately large relative to neighbouring years from the same institution, and also when compared to other institutions, that their reliability and plausibility came into question. As our goal was to understand overall trends in global collecting activities across these taxa, and these anomalies represented implausible data that impaired our ability to analyse high level trends and inferences, we chose to implement an anomaly filtering process.<br><br>In our analysis, we employed conservative thresholds to identify and remove a small number of anomalous datasets while maximising the overall data retained. We chose thresholds based on standard deviations (SDs) on the log scale compared to the counts in that collection year from other institutions, and also relative to counts in all collection years for that institution. The use of standard deviations on the log scale to detect outliers is a common practice rooted in statistical theory4,5. This was designed as a cautious approach, and thresholds were explicitly chosen through iterative testing to remove a small fraction of records that seemed clearly anomalous and implausible, while retaining as much data as possible to support our main analyses. As addressed in the Discussion, this was not designed or intended to be an exhaustive process, and there are likely other instances of data quality issues remaining to be resolved at the institutional or aggregator level. This includes GBIF's ongoing quality assurance processes which are already in place, as evident in the instances of records being removed at several junctures over time, seen from the historical GBIF database snapshots (Supplementary Figures 10-12).<br><br>The steps involved in this anomaly filtering process were as follows:<br>1. Log Transformation: We applied a log transformation to the yearly counts to stabilise variance, given large variation on the linear scale, allowing similar scale thresholds to be applied across taxa.<br>2. Standard Deviation Calculation: For each institution, we calculated the mean and standard deviation of the log-transformed counts.<br>3. Threshold Application: For contemporary records, institution-level datasets that exceeded 3 standard deviations above the mean log-transformed count (both relative to all years for that institution, and relative to all other institutions in that collection year) were flagged as anomalous. This corresponded to a range of 16.9 – 104.6 linear scale standard deviations above the mean annual count across institutions, representing an extremely large departure from the typical range. Based on iterative testing, more conservative thresholds of 3.1 log SDs (Plantae and Chordata) and 3.6 log SDs (Arthropoda) were used for anomaly removal in historical snapshots, due to greater variability in the magnitude of institution-level annual record counts in the historical snapshots data.<br>4. Comparison Across Years & Institutions: These flagged datasets were further inspected and compared against counts from other collection years and other institutions to assess plausibility and reliability.<br>5. Removal of Anomalous Records: Finally, records identified as anomalous through these stringent criteria were removed from the dataset to maintain data integrity, and we contacted the contributing institutions for these datasets to identify possible causes for the data anomalies. Of the 6 institutions contacted, 1 responded (INSDC EMBL-EBI), who confirmed that their uploads to GBIF duplicated entries from other contributing institutions including CBG Guelph, another institution whose records were also flagged as anomalous – for Arthropoda data in Canada between 2012 – 2015.<br><br>Further details on data cleaning are provided in the Supplementary Information, Sections 3 - 5.
Reproducibility	All data are downloadable directly from GBIF and Zenodo, and all of our code is provided with clear documentation to enable reproducibility and transparency in our analyses.
Randomization	NA
Blinding	NA

Did the study involve field work? ☐ Yes ☒ No

Reporting for specific materials, systems and methods

We require information from authors about some types of materials, experimental systems and methods used in many studies. Here, indicate whether each material, system or method listed is relevant to your study. If you are not sure if a list item applies to your research, read the appropriate section before selecting a response.

Materials & experimental systems

n/a	Involved in the study
☒	☐ Antibodies
☒	☐ Eukaryotic cell lines
☒	☐ Palaeontology and archaeology
☒	☐ Animals and other organisms
☒	☐ Clinical data
☒	☐ Dual use research of concern
☒	☐ Plants

Methods

n/a	Involved in the study
☒	☐ ChIP-seq
☒	☐ Flow cytometry
☒	☐ MRI-based neuroimaging

Plants

Seed stocks	NA
Novel plant genotypes	NA
Authentication	NA
